# Drug-induced liver injury in association with antipsychotics

**DOI:** 10.1192/j.eurpsy.2022.1885

**Published:** 2022-09-01

**Authors:** R. Zeiss, M. Gahr

**Affiliations:** University of Ulm, Department Of Psychiatry, Ulm, Germany

**Keywords:** hepatotoxicity, Antipsychotics, pharmacovigilance, drug-induced liver injury

## Abstract

**Introduction:**

Drug-induced liver injury is one of the leading causes for acute liver failure and drug withdrawal after marketing approval. One important risk factor is the extent of exposure of the hepatocytes to a substance, either by high doses or by long-term medication. In many psychiatric diseases, like schizophrenia long-term use of drugs is common. However, systematic data on the hepatotoxic potential of antipsychotics is scarce.

**Objectives:**

To perform an explorative analysis of pharmacovigilance data on the risk of hepatotoxicity related to the use of antipsychotics.

**Methods:**

We conducted an explorative case/non-case study based on data from VigiBase for 30 antipsychotics marketed in the European Union. Reporting odds ratios were calculated for antipsychotics associated with the SMQ “Drug related hepatic disorders - comprehensive search” and the SMQ “Drug related hepatic disorders - severe events only”.

**Results:**

We found several associations of antipsychotics with drug-induced liver injury including associations with severe events. 17/30 antipsychotics were associated with “Drug related hepatic disorders - comprehensive search”, and for 10/30 substances were associated with severe hepatic events.

**Conclusions:**

Several antipsychotics are associated with the risk for hepatotoxic side effects, even severe ones. Further research is warranted on patient and substance-dependent risk factors.

**Disclosure:**

No significant relationships.

